# The Role, Interaction and Regulation of the *Velvet* Regulator VelB in *Aspergillus nidulans*

**DOI:** 10.1371/journal.pone.0045935

**Published:** 2012-09-25

**Authors:** Hee-Soo Park, Min Ni, Kwang Cheol Jeong, Young Hwan Kim, Jae-Hyuk Yu

**Affiliations:** 1 Department of Bacteriology and Genetics, University of Wisconsin, Madison, Wisconsin, United States of America; 2 Department of Molecular and Environmental Toxicology Center, University of Wisconsin, Madison, Wisconsin, United States of America; 3 Mass Spectrometry Research Center/Mass Spectrometry Team, Korea Basic Science Institute, Daejeon, Republic of Korea; University of Nebraska, United States of America

## Abstract

The multifunctional regulator VelB physically interacts with other *velvet* regulators and the resulting complexes govern development and secondary metabolism in the filamentous fungus *Aspergillus nidulans*. Here, we further characterize VelB’s role in governing asexual development and conidiogenesis in *A. nidulans*. In asexual spore formation, *velB* deletion strains show reduced number of conidia, and decreased and delayed mRNA accumulation of the key asexual regulatory genes *brlA*, *abaA*, and *vosA*. Overexpression of *velB* induces a two-fold increase of asexual spore production compared to wild type. Furthermore, the *velB* deletion mutant exhibits increased conidial germination rates in the presence of glucose, and rapid germination of conidia in the absence of external carbon sources. *In vivo* immuno-pull-down analyses reveal that VelB primarily interacts with VosA in both asexual and sexual spores, and VelB and VosA play an inter-dependent role in spore viability, focal trehalose biogenesis and control of conidial germination. Genetic and *in vitro* studies reveal that AbaA positively regulates *velB* and *vosA* mRNA expression during sporogenesis, and directly binds to the promoters of *velB* and *vosA*. In summary, VelB acts as a positive regulator of asexual development and regulates spore maturation, focal trehalose biogenesis and germination by interacting with VosA in *A. nidulans.*

## Introduction

Fungal spores are widespread in the environment and have a significant impact on daily human life. Asexual spores are the main reproductive form of many fungi and the primary agent for infecting hosts for many pathogenic fungi [1]. In some *Aspergillus,* asexual spore formation is closely correlated with the production of toxic secondary metabolites called mycotoxins [2], [3]. Among *Aspergillus* species, *Aspergillus nidulans* is an excellent model system for studying the regulation of gene expression and the mechanisms of asexual development [4], [5].

**Table 1:** *Aspergillus* strains used in this study.

Strain name	Relevant genotype	References
FGSC4	*A. nidulans* wild type, *veA^+^*	FGSC a
FGSC26	*biA1*; *veA1*	FGSC a
FGSC33	*biA1*; *pyroA4*; *veA1*	FGSC a
RJMP1.59	*pyrG89*; *pyroA4*; *veA* ^+^	N. P. Keller
FGSC583	*biA1*; *brlA42*; *veA1*	[52]
FGSC590	*biA1*; *abaA14*; *veA1*	[52]
FGSC580	*biA1*; *wetA6*; *veA1*	[52]
TTA292-1	*biA1*; *argB*::*alcA*(p)::*brlA*; *metG1*; *veA1*	[48]
SJA7	*pabaA1*, *yA2*; *pyroA4*; *alcA*(p)::*abaA*; *veA1*	J. Aguirre
TTA021	*biA1*, *pabaA1*; *alcA*(p)::*brlA*; *abaA14*; *veA1*	[48]
RNI14.1	*biA1*; Δ*vosA*::*argB* ^+^; *veA* ^+^	[13]
RNI18.3	*pyroA4*; *ΔvelB*::*AfupyrG* ^ +^; *veA* ^+^	[15]
TNJ140.1	*pyrG89*; *pyroA4*; Δ*ganB*::*AfupyrG* ^ +^; *veA* ^+^	N.-J. Kwon & J.-H. Yu
THS13.1	*pyroA4*; Δ*ganB*::*pyroA^+^*, Δ*velB*::*AfupyrG* ^ +^; *veA* ^+^	This Study
THS14.1	*pyroA4*; Δ*vosA*::*pyroA^+^*; Δ*velB*::*AfupyrG* ^ +^; *veA* ^+^	This Study
THS16.1	*pyrG89*; *pyroA4*; Δ*velB*::*AfupyrG* ^ +^; *veA* ^+^	This Study
THS20.1	*pyrG89*; *pyroA*::*velB*(p)::*velB*::FLAG_3x_::*pyroA* b; Δ*velB*::*AfupyrG* ^ +^; *veA* ^+^	This Study
THS28.1	*pyrG89*; *pyroA*::*vosA*(p)::*vosA*::FLAG_3x_::*pyroA* b; Δ*vosA*::*AfupyrG* ^ +^; *veA* ^+^	This Study
THS7.1	*biA1*; *pyroA*::*alcA*(p)::*velB*::FLAG::*pyroA* b; *veA1*	This Study
THS18.1	*pyrG89*; *pyroA*::*alcA*(p)::*velB*::FLAG::*pyroA* b; *veA* ^+^	This Study
RHS2.1,2	*pyroA*::*alcA*(p)::*velB*:: FLAG::*pyroA* b; *veA1*	This Study
RHS2.3,4	*pyroA*::*alcA*(p)::*velB*:: FLAG::*pyroA* b; *veA* ^+^	This Study

a Fungal Genetic Stock Center.

b The 3/4 *pyroA* marker selects for the targeted integration at the *pyroA* locus.

Asexual development (conidiation) in *A. nidulans* involves the formation of specialized multicellular structures called conidiophores. Conidiophore formation starts from hyphal cells, which form the mycelium, followed by the sequential formation of vesicles, metulae, phialides and conidia [4], [6]. The process of conidiation is genetically regulated and the three genes *brlA*, *abaA* and *wetA* have been proposed to define a central regulatory pathway activating conidiophores formation [4], [7]–[10]. After the spore is physically formed, it must undergo the maturation process. During conidiophore maturation, a key process is the focal biogenesis of large amounts (up to 15% of dry weight) of trehalose, α-D-glucopyranosyl-α-D-glucopyranoside, within the spore thereby conferring long term viability [11]. Trehalose, found in a wide variety of bacteria, fungi, plant, and invertebrates, serves as a vital protectant against desiccation and various environmental stresses and as an energy source [12]. Recent studies have identified the novel regulator VosA, which functions in trehalose biosynthesis and conidia maturation in *A. nidulans*. VosA also exerts negative feedback control of conidiation by down-regulating *brlA* expression. Moreover, VosA is mainly localized in the nucleus of mature conidia and it contains a potential transcriptional activation domain at the C-terminus. These findings led to the hypothesis that VosA is a transcription factor regulating conidia maturation and the completion of conidiogenesis [13].

The *velvet* family proteins, including VeA, VelB, VelC and VosA, have defined a novel protein family. These four proteins all contain the *velvet* domain, and are highly conserved in dimorphic and filamentous fungi [13], [14]. A series of recent studies have revealed that the *velvet* proteins form the multimeric *velvet* complexes such as VelB/VeA/LaeA, VelB/VosA, and VelB/VelB [15], [16]. The heterotrimeric VelB/VeA/LaeA complex controls sexual development and secondary metabolism in response to light [15]. The nuclear VelB-VosA heterodimer has been proposed to regulate trehalose biogenesis and spore maturation [16].

**Table 2:** Oligonucleotides used in this study.

Name	Sequence (5′ 3′)	Purpose
OJA142	CTGGCAGGTGAACAAGTC	5′ *brlA* probe
OJA143	AGAAGTTAACACCGTAGA	3′ *brlA* probe
OJA154	AGCTCTTCAGAATACGTC	5′ *abaA* probe
OJA155	GTTGTGAGATGCCTCCAT	3′ *abaA* probe
OMN66	TTTCCAGATCCTTCGCAG	5′ *vosA* probe
OMN63	ATAGAAACAGCCACCCAG	3′ *vosA* probe
OMN125	TATGCACTGGCACTCAAGCAACCG	5′ *velB* probe
OMN126	GTGCATGACGGTCGTATCTGGTCC	3′ *velB* probe
OMN176	CCATCACCATAAAGCGATCAG	5′*tpsA* probe
OMN177	CAGTTTCGAGAAGTTAAGCGC	3′*tpsA* probe
OMN338	CATGGCCACGATACAATCCTAG	5′ *rosA* probe
OMN339	AAGATAGTGCCATTCCCGAGCC	3′ *rosA* probe
OHS490	ATGCCGGCAGCACCGAGAAAG	5′ *nosA* probe
OHS491	TCAAAGAAGAAGGTAGTTCCAACCG	3′ *nosA* probe
OMN340	ATGGGATCACTAGAGGCTGGAC	5′ *nsdD* probe
OMN341	TTAATGACTCCTCGGTGACACC	3′ *nsdD* probe
OJA246	CAGCCTCAAGCAAGCAACTGA	5′ *mutA* probe
OJA247	CTAGTTGACATAATCGCTTGG	3′ *mutA* probe
OJH84	GCTGAAGTCATGATACAGGCCAAA	5′ *AfupyrG* marker
OJH85	ATCGTCGGGAGGTATTGTCGTCAC	3′ *AfupyrG* marker
OMN131	GAAGGTCGATGATGGTGTGATG	5′ flanking region of *velB*
OMN132	CTAGAGGTAAAGATCAAGGTAG	3′ flanking region of *velB*
OMN133	CTGATGGCTGAATGAAGCACAG	5′ nested of *velB*
OMN134	TGCTTTACGACGATAGCCATGC	3′ nested of *velB*
OMN135	*GGTGAAGAGCATTGTTTGAGGCA* GCGGCCAGTCTTTAGACAAATG	5′ *velB* with *AfupyrG* tail
OMN136	*AGTGCCTCCTCTCAGACAGAATA* GGATAACGAATACTAAAGACCG	3′ *velB* with *AfupyrG* tail
OMN54	TTTTTGCCGCTGCTGGAGTTAG	5′ flanking region of *vosA*
OMN55	AAGAGGGCTTTGTGGGGTTTTC	3′ flanking region of *vosA*
OMN58	GCTATAACAAAGAGAGAGAGGG	5′ nested of *vosA*
OMN59	TTCGAAAAATATGCCGGGGCTG	3′ nested of *vosA*
OHS184	*ACTTCTGCAGTCGGAATTGGCCTG* GAGCACTATGAGAGACGACTG	5′ *vosA* with *pyroA* tail
OHS185	*TGGTGAGAACACATGCACAACTTG* GGATTCTCGTTTGTGGAACAC	3′ *vosA* with *pyroA* tail
OHS170	ACCCACGGTCAACTGAGAAGATG	5′ flanking region of *ganB*
OHS171	TGCGGAGGAGCAGTTGTCAGTG	3′ flanking region of *ganB*
OHS172	AGCTGTCTTAGATTCGGTGCGGG	5′ nested of *ganB*
OHS173	GGAGAGACAGCTCACATGGCACTG	3′ nested of *ganB*
OHS174	*ACTTCTGCAGTCGGAATTGGCCTG* AGATACTGCCTTCGACAGCGTCC	5′ *ganB* with *pyroA* tail
OHS175	*TGGTGAGAACACATGCACAACTTG* GACAACTGAGTCGCCTTAGCCTC	3′ *ganB* with *pyroA* tail
ONK395	ATCTCATGGGTGCTGTGCGAAAGG	5′ *pyroA* marker
ONK396	TTGCATCGCATAGCATTGCATTGC	3′ *pyroA* marker
OMN196	CG**GGATCC**ATGTACGCTGTTGAGGATAGG	5′ *velB* with BamHI
OHS140	AATT**GAATTC**GTAGGTGGTGTACGCGATTGACGG	5′ *velB* with EcoRI
OHS141	AATT**AAGCTT**GTATTCGTTATCCAGACCATCGTCCTC	3′ *velB* with HindIII
OHS258	AATT**GAATTC**CCGTGATGACCCTCTACAGCATTAC	5′ *abaA* with EcoRI
OHS259	AATT**GCGGCCGC**TTAATGAGATGGTTGATAGAGGGACCAAG	5′ *abaA* with NotI
OMN364	CAGCTCCATTCCGCACGGTCATTCC	5′ *velB* EMSA probe 1
OMN365	GGAATGACCGTGCGGAATGGAGCTG	3′ *velB* EMSA probe 1
OHS408	AGCTCCAGCTCCATTCCGCACGGTCATTCCGTTCTCGGCG	5′ *velB* EMSA probe 2
OHS409	CGCCGAGAACGGAATGACCGTGCGGAATGGAGCTGGAGCT	3′ *velB* EMSA probe 2
OHS395	GTGGTTCCTTACCTCATTCCCGACCTTTTTTGCC	5′ *vosA* EMSA probe 3
OHS396	GGCAAAAAAGGTCGGGAATGAGGTAAGGAACCAC	3′ *vosA* EMSA probe 3
OHS148	CAGCTCCAAACCGCACGGTCATTCC	5′ *velB* EMSA probe M1
OHS149	GGAATGACCGTGCGGTTTGGAGCTG	3′ *velB* EMSA probe M1
OHS414	AGCTCCAGCTCCAAACCGCACGGTCAAACCGTTCTCGGCG	5′ *velB* EMSA probe M2
OHS415	CGCCGAGAACGGTTTGACCGTGCGGTTTGGAGCTGGAGCT	3′ *velB* EMSA probe M2
OHS397	GTGGTTCCTTACCTCAAACCCGACCTTTTTTGCC	5′ *vosA* EMSA probe M3
OHS398	GGCAAAAAAGGTCGGGTTTGAGGTAAGGAACCAC	3′ *vosA* EMSA probe M3

a Tail sequence is in italic.

b Restriction enzyme site is in bold.

c Mutagenized site is underlined.

VelB, a primary component of the *velvet* complexes, plays a crucial role in sexual development and secondary metabolism in *A. nidulans* [15], [16]. However, additional role and interaction of VelB in the life cycle of conidiogenesis, and temporal regulation of *velB* expression in the late phase of conidiation in *A. nidulans* have not been studied. In this study, we further characterize *velB* by genetic and biochemical approaches. The deletion of *velB* results in elevated accumulation of brown pigment(s) and decreased production of conidia. Conversely, overexpression of *velB* results in increased conidiation in air-exposed conditions, suggesting an activating role of VelB in conidiation. In addition, VelB plays a negative role in regulating conidial germination regardless of the presence or absence of an external carbon source. VelB predominantly interacts with VosA in asexual and sexual spores and plays an inter-dependent role in focal trehalose biogenesis, conidial maturation and germination. We further demonstrate that AbaA positively regulates *velB* and *vosA* mRNA expression during the mid-to-late phase of conidiation and directly binds to the promoters of these genes. Taken together, we present a model that VelB acts as a positive regulator of conidiation and AbaA activates expression of VelB and VosA in phialides, which then primarily forms the VelB/VosA heterodimer in conidia and controls focal trehalose biosynthesis and conidial germination.

**Figure 1:**
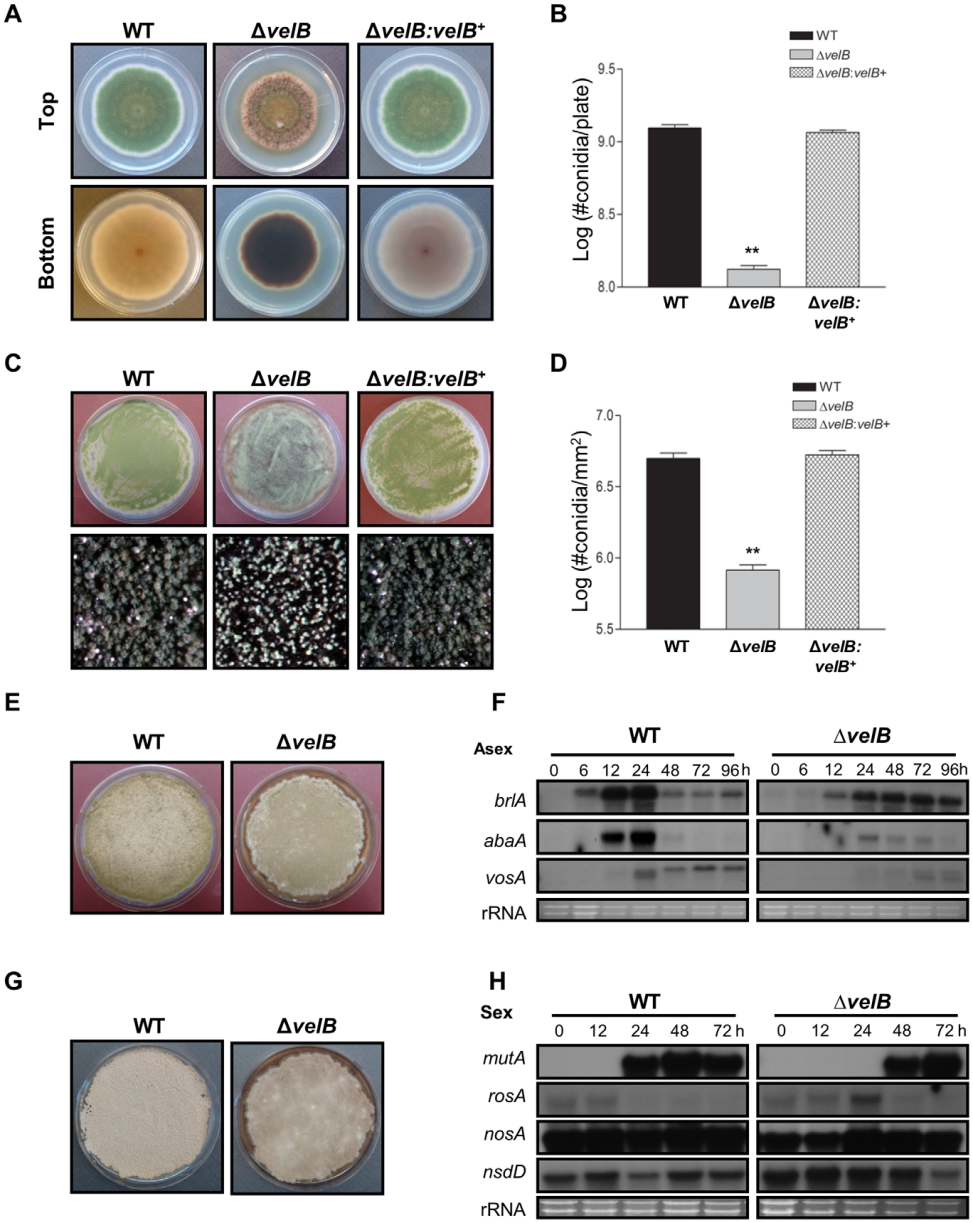
Phenotypes of the Δ*velB* mutant. (A) Colony photographs of WT (FGSC4), Δ*velB* (THS16.1) and complemented (THS20.1) strains point-inoculated on solid MM and grown for 4 days (Top and bottom panels). The bottom panel shows the underside of the plates. (B) Quantitative analysis of conidiation of strains shown in (A) (** P<0.05). (C) About 10^5^ conidia of WT (FGSC4), Δ*velB* (THS16.1) and complemented (THS20.1) strains were spread onto solid MM and grown for 2 days. The bottom panel shows close-up views of the middle of the plates. (D) Quantitative analysis of conidiation of strains shown in (C) (** P<0.05). (E) Phenotypes of WT and Δ*velB* strains post asexual developmental induction. (F) Northern blot for *brlA*, *abaA* and *vosA* mRNA in WT (FGSC4) and Δ*velB* (THS16.1) strains post asexual developmental induction (Asex). Numbers indicate the time (h) of incubation after induction of asexual development. Equal loading of total RNA was confirmed by ethidium bromide staining of rRNA. (G) Phenotypes of WT and Δ*velB* strains post sexual developmental induction. (H) Northern blot for *mutA, rosA, nosA* and *nsdD* mRNA levels in WT (FGSC4) and Δ*velB* (THS16.1) strains post sexual developmental induction (Sex). Numbers indicate the time (h) of incubation after induction of sexual development. Equal loading of total RNA was confirmed by ethidium bromide staining of rRNA.

## Materials and Methods

### Strains, Media and Culture Conditions

*A. nidulans* strains used in this study are listed in Table 1. The fungal strains were grown on solid or liquid minimal medium with supplements (simplified as MM) as described previously [13], [17] and incubated at 37°C. To determine the number of conidia, wild type (WT) and mutant strains were point inoculated and grown on solid MM at 37°C for 3 to 5 days. The conidia were collected in ddH_2_O from the entire colony and counted using a hemocytometer. For liquid submerged cultures, conidia of WT and mutant strains were inoculated in 50 ml of liquid MM (5×10^5^ conidia/ml) and incubated at 37°C, 250 rpm. To examine the effects of overexpression (OE) of *velB* by an ectopic copy of *velB* under the *alcA* promoter, all strains were inoculated on solid MM with 1% glucose (MMG) or MM with 100 mM threonine (MMT to induce overexpression of *velB*) and 0.1% yeast extract at 37°C for 4 days.

For Northern blot analysis, samples were collected as described [13], [18], [19]. Briefly, for vegetative growth phases, conidia (5×10^5^ conidia/ml) of WT and mutant strains were inoculated in 100 ml liquid MM in 500 ml flasks and incubated at 37°C. Samples were collected at designated time points of liquid submerged culture, squeeze-dried and stored at −80°C. For sexual and asexual developmental induction, 18 h vegetatively grown mycelia were filtered, washed and transferred to solid MM and the plates were air exposed for asexual developmental induction, or tightly sealed and blocked from light for sexual developmental induction. To examine *velB* mRNA levels in *alcA*(p)::*brlA*, *alcA*(p)::*brlA abaA14* and *alcA*(p)::*abaA* strains, three strains were grown in liquid glucose medium (G) at 37°C, 250 rpm for 14 h (designated as time point “0”) and then transferred into liquid glucose medium (G; non-inducing) or liquid threonine medium (T; inducing). Samples were collected at designated time points after transfer. *Escherichia coli* strains, DH5α and BL21 (DE3), were grown in Luria–Bertani medium with ampicillin (50 mg/ml) for plasmid amplification.

**Figure 2:**
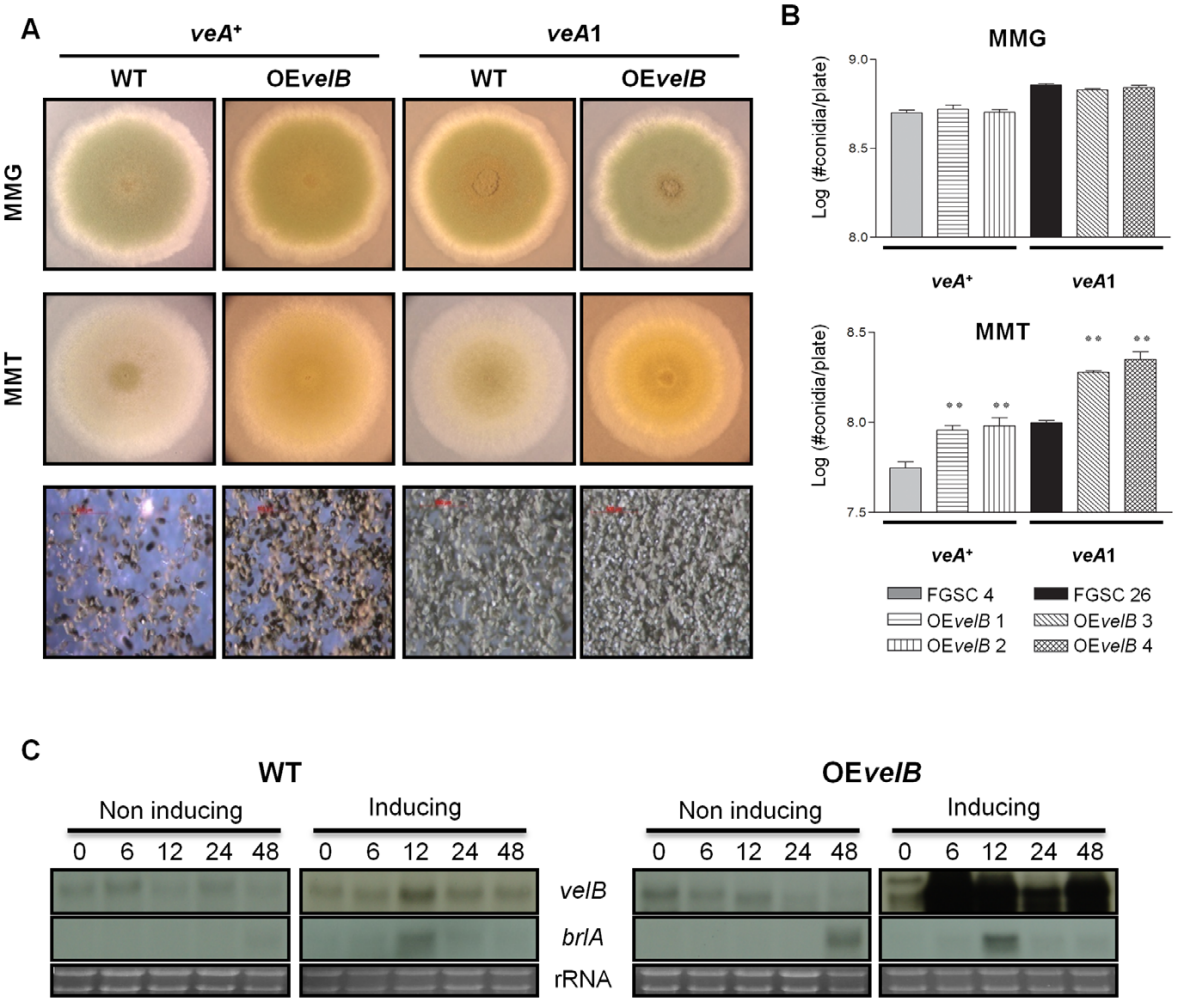
Effects of overexpression of *velB*. (A) WT (FGSC4: *veA+* and FGSC26: *veA1*) and *velB* overexpression (RHS 2.1 and RHS2.3) strains were point inoculated on solid MMG (non-inducing) or MMT (100 mM threonine, inducing) and photographed at day 4. (B) Effects of overexpression of *velB* in conidiospore formation. Quantification was done as described in the experimental procedures (** P<0.05). (C) Northern blot for mRNA levels of *brlA* in WT (FGSC4) and OE*velB* (RHS 2.1) strains in liquid submerged culture. Numbers indicate the time (h) post transfer to liquid MMG (non-inducing) or liquid MMT (inducing). Equal loading of total RNA was confirmed by ethidium bromide staining of rRNA.

### Generation of *velB* Mutants

The oligonucleotides used in this study are listed in Table 2. For the deletion of *velB*, DJ-PCR method was used [20]. Both flanking regions of *velB* were amplified using the primer pairs OMN131;OMN135 and OMN132;OMN136 using *A. nidulans* FGSC4 genomic DNA as a template. The *A. fumigatus pyrG* marker was PCR-amplified from *A. fumigatus* AF293 genomic DNA with the primer pair OJH84;OJH85. The final PCR constructs for the *velB* deletion were amplified with OMN133;OMN134. The deletion cassettes were introduced into RJMP1.59 protoplasts generated by the Vinoflow FCE lysing enzyme (Novozymes) [21]. To generate the double deletion mutants, 5′ and 3′ flanking regions of *vosA* (OMN54;OHS184 and OMN55;OHS185) and *ganB* (OHS170;OHS174 and OHS171;OHS175) were amplified. The *pyroA* marker was amplified from FGSC4 genomic DNA with the primer pair ONK395;ONK396. After the fusion by DJ-PCR, *vosA* and *ganB* deletion constructs were amplified using OMN58;OMN59 and OHS172;OHS173, respectively, and introduced into RNI 18.3. Multiple (at least three) double mutants were isolated and confirmed by PCR followed by restricted enzyme digestion in each case.

**Figure 3:**
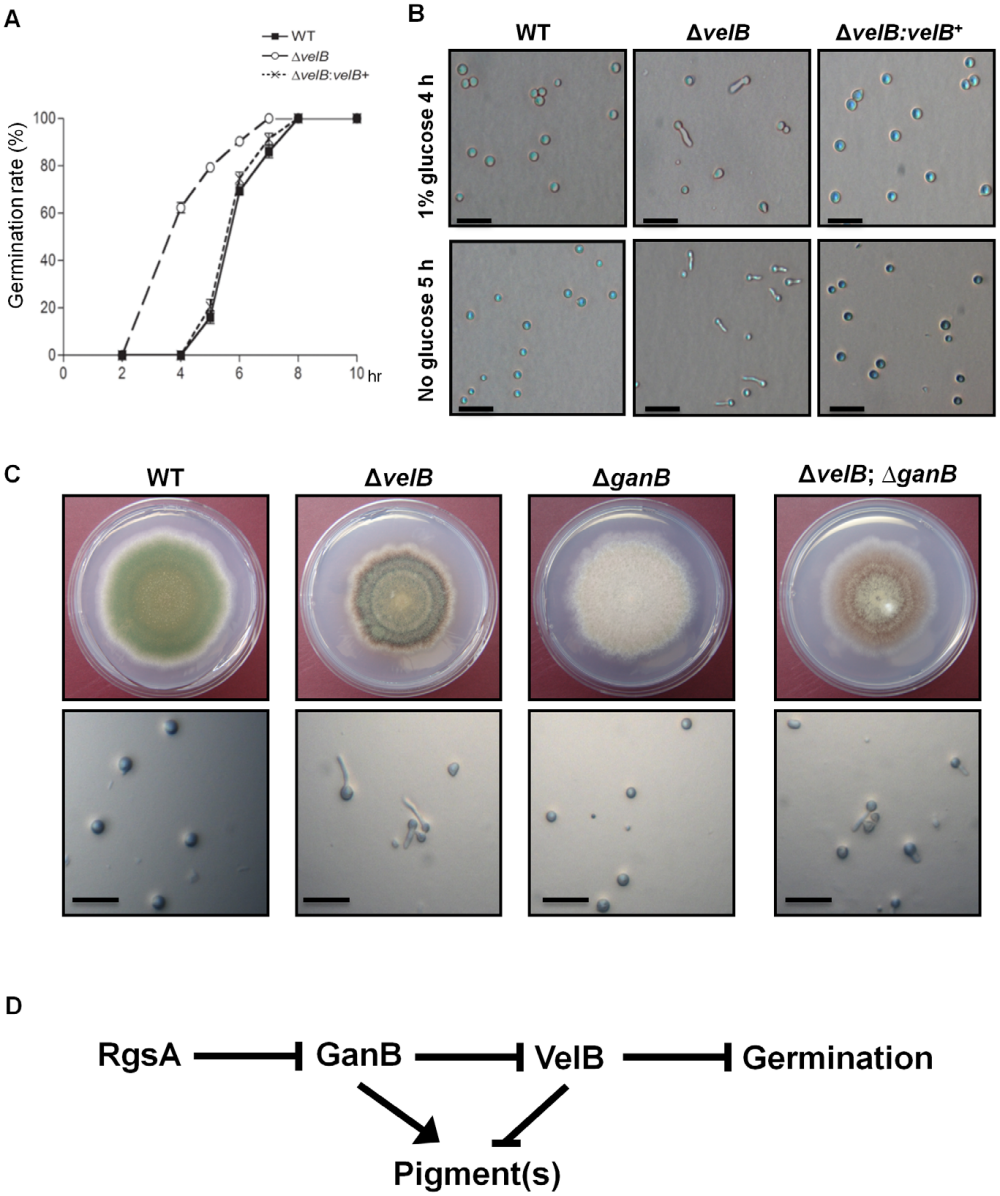
Roles of *velB* in conidial germination. (A) Kinetics of germ tube formation in inoculated WT (FGSC4), Δ*velB* (THS16.1) and complemented (THS20.1) strains inoculated on solid MM at 37°C in the presence of glucose (1%). The number of conidia showing a germ tube protrusion was recorded at different times and is presented as a percentage of the total number of conidia in these fields. Data were obtained from two independent experiments. (B) Conidia of the designated strains were inoculated on solid MM (1% glucose) with or without carbon source and incubated at 37°C for 4∼5 h (bar = 0.02 mm). (C) Colonies of WT (FGSC4), Δ*velB* (THS16.1), Δ*ganB* (TNJ140.1) and Δ*velB* Δ*ganB* (THS13.1) strains grown on solid MM for 4 day (Top panels). Status of conidial germination of the same strains on solid MMG, 37°C for 4 h (Bottom panels, bar = 0.02 mm). (D) Proposed model of GanB and VelB in controlling pigmentation and conidial germination.

To generate the *alcA*(p)::*velB* fusion construct, the *velB* ORF derived from genomic DNA was PCR amplified using the primer pair OMN196;OHS141. The PCR product was then double digested with *Bam*HI and *Hin*dIII and cloned into pHS3, which contains the *A. nidulans alcA* promoter and the *trpC* terminator (this study). The resulting plasmid pHSN6 was then introduced into RJMP1.59 or FGSC 33. The *velB* overexpression strains among the transformants were screened by Northern blot analysis using a *velB* ORF probe followed by PCR confirmation. RHS2.1, 2.2, 2.3 and 2.4 were isolated from the sexual cross between THS7.1 and THS18.1.

To complement Δ*velB*, the WT *velB* gene region including its predicted promoter was amplified with the primer pair OHS140;OHS141, digested with *Eco*RI and *Hin*dIII and cloned into pHS13. This cloning vector contains the ¾*pyroA* gene [22], a 3xFLAG tag and the *trpC* terminator [23]. The resulting plasmid pHSN31 was then introduced into the recipient Δ*velB* strain THS16.1, where preferentially a single copy *velB^+^* gets inserted into the *pyroA* locus, to give rise to THS20.1.

**Figure 4:**
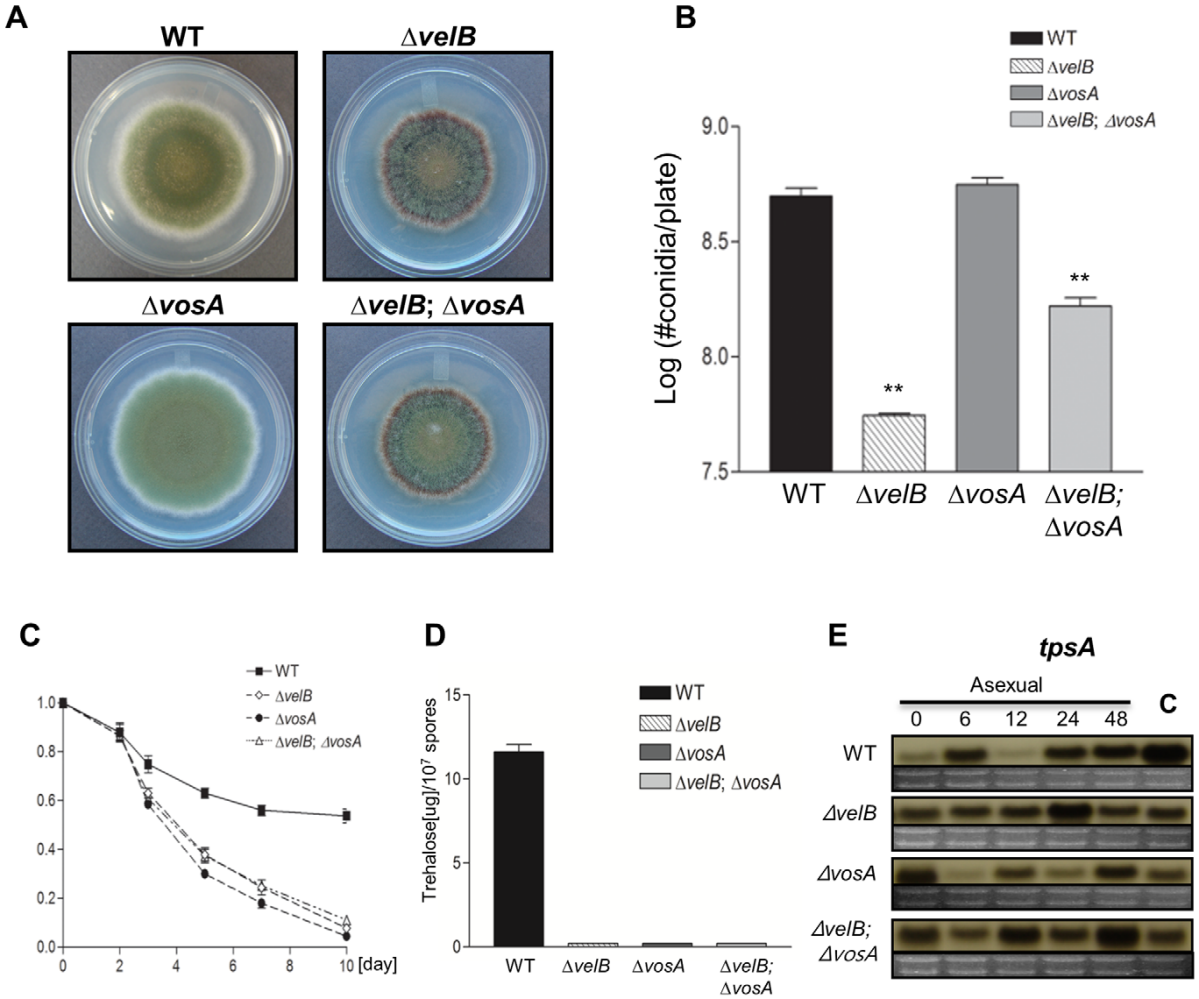
VelB and VosA are inter-dependent for sporogenesis and trehalose biogenesis in spores. (A) Colony photographs of WT (FGSC4), Δ*velB* (THS16.1), Δ*vosA* (RNI14.1) and Δ*velB* Δ*vosA* (THS14.1) strains grown on solid MM for 4 days. (B) Quantitative analysis of conidiation of strains shown in (A) (** P<0.05). (C) Viability of conidia collected from the designated strains grown at 37°C for 2, 3, 5, 7, and 10 days. (D) The amount of trehalose (µg) per 10^7^ conidia from the 2 day old colonies of the designated strains (triplicates measurements). (E) Levels of *tpsA* transcript in the designated strains post asexual developmental induction (A). C = conidia. Equal loading of total RNA was confirmed by ethidium bromide staining of rRNA.

### Nucleic Acid Manipulation

To isolate genomic DNA, about 10^6^ conidia of WT and mutant strains were inoculated in 2 ml liquid MMG +0.5% yeast extract, and stationary cultured at 37°C for 24 h. The mycelial mat was collected, squeeze-dried, and genomic DNA was isolated as described [20]. Total RNA isolation and Northern blot analyses were carried out as previously described [18], [20], [24]. The DNA probes were prepared by PCR-amplification of the coding regions of individual genes with appropriate oligonucleotide pairs using FGSC4 genomic DNA as a template (Table 2).

### Spore Viability Test

Spore viability was determined as described [13]. Two-day old conidia (10^5^ per plate) of WT and mutant strains were spread onto solid MM and incubated at 37°C. After 2∼10 days, the conidia were collected and counted using a hemocytometer. Approximately 200 conidia were spread onto solid MM and incubated for 2 days at 37°C in triplicates. Survival rates were calculated as a ratio of the number of viable colonies to the number of spores inoculated.

**Figure 5:**
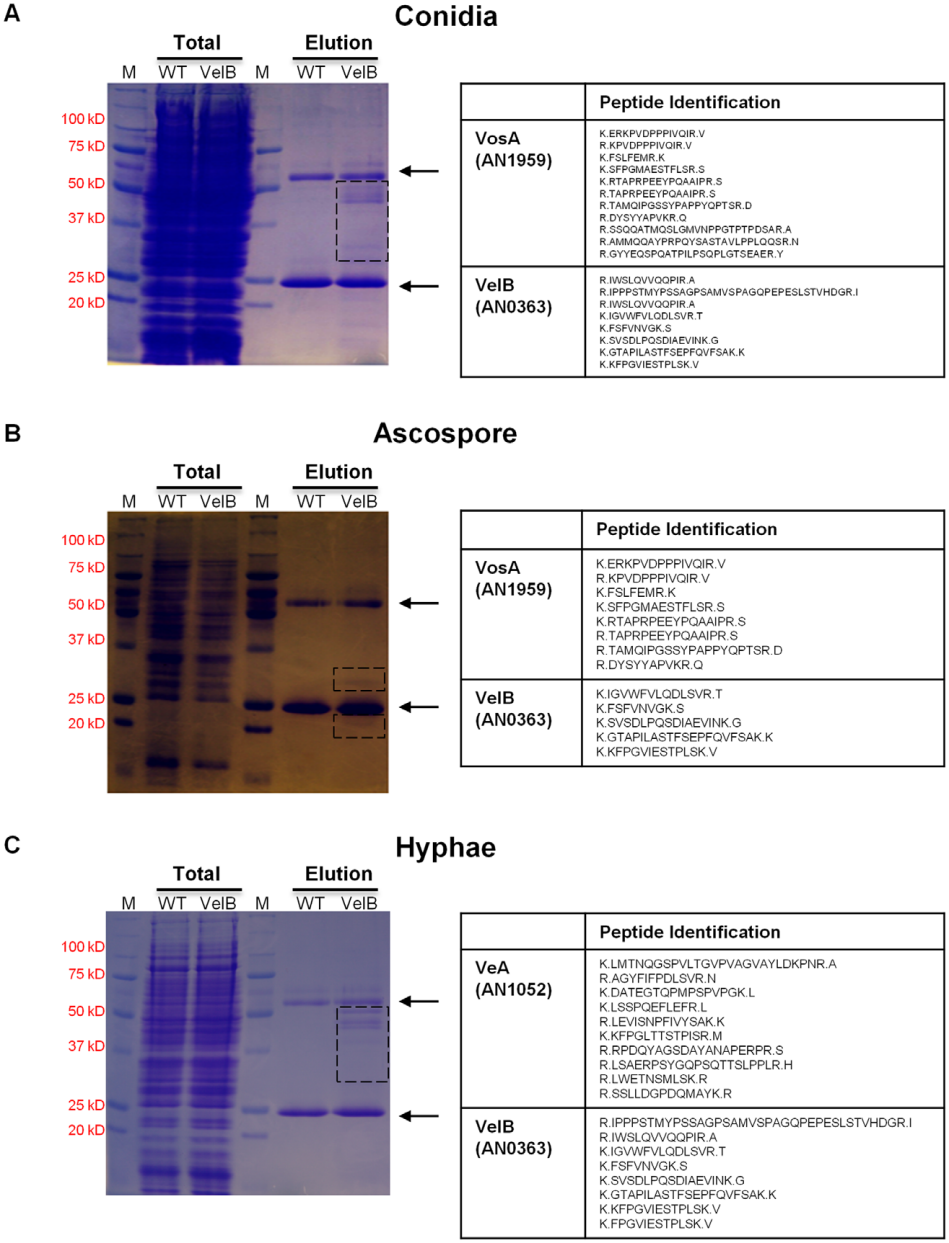
*In vivo* interacting proteins of VelB in conidia, ascospores and hyphae. (A)∼(C) SDS-polyacrylamide (10%) gel electrophoresis of total and VelB-FLAG enriched proteins stained with brilliant blue G. Lanes 1 & 4: Molecular mass standard; lane 2: soluble lysates from WT strain; lane 3: soluble lysates from THS 20.1 (Δ*velB*; *velB*::3xFLAG) strain; lane 5: Eluted proteins from WT strain; lane 6: Eluted proteins rom THS 20.1 (Δ*velB*; *velB*::3xFLAG) strain. Arrows indicate the antibody fragments. After staining, the indicated regions were excised and proteins were identified using a nano-LC-MS system. Polypeptides identified from the bands of the VelB-FLAG affinity purification from conidia (A), ascospores (B) and hyphae (C) are shown.

### Spore Trehalose Assay

Trehalose was extracted from conidia and analyzed as described [13], [25]. Two-day old conidia (2×10^8^) were collected and washed with ddH_2_O. Conidia were resuspended in 200 µl of ddH_2_O and incubated at 95°C for 20 min, and then the supernatant was collected by centrifugation. The supernatant was mixed with an equal volume of 0.2 M sodium citrate (pH5.5) and further incubated at 37°C for 8 h with or without (control) 3 mU of trehalase (Sigma), which hydrolyzes trehalose to glucose. The amount of glucose generated by trehalose was assayed with a glucose assay kit (Sigma), and converted into the trehalose amount per conidium (triplicates). Each sample not treated with trehalase served as a negative control.

### Germination of Conidia

Germination rates were measured as previously described [26]. The conidia of WT and mutants were spread onto solid MM containing glucose or no carbon source and incubated at 37°C. Levels of germination (isotropic growth and germ tube formation) were examined every 1 h after inoculation under a microscope.

**Figure 6:**
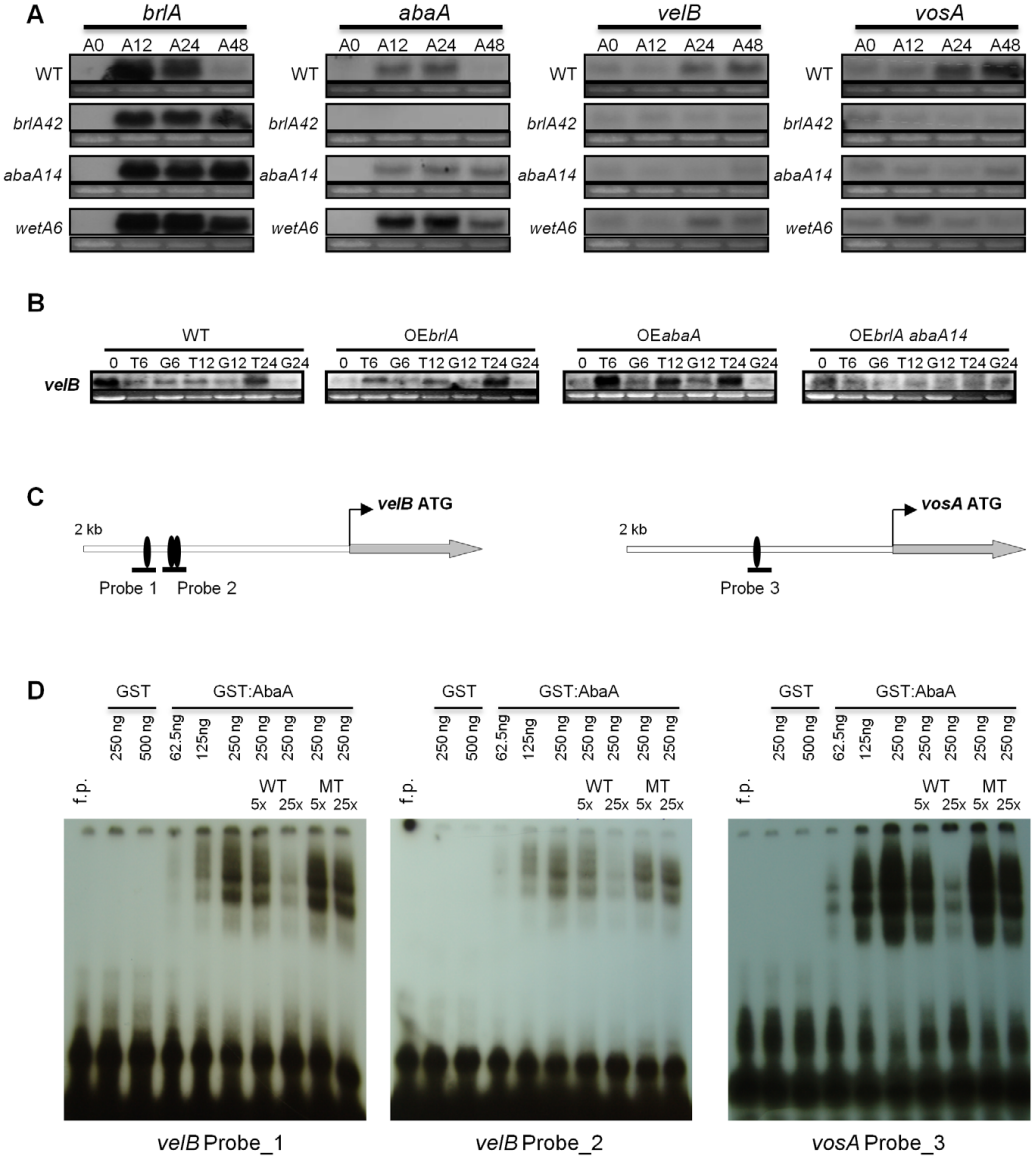
AbaA regulates *velB* and *vosA* expression. (A) Northern blot analyses for the levels of *velB*, *brlA*, *abaA* and *vosA* transcripts in WT (FGSC26), *brlA42* (FGSC A583), *abaA14* (FGSC A590), and *wetA6* (FGSC A580) strains at 0, 12, 24, 48 h of asexual development (A0∼A48). *brlA42*, *abaA14* and *wetA6* are temperature sensitive alleles that exhibit loss of function at 37°C. (B) Northern blot analyses for the levels of *velB* mRNA in WT (FGSC26), *alcA*(p)::*brlA* (TTA292-1), *alcA*(p)::*abaA* (SJA7) and *alcA*(p)::*brlA abaA14* (TTA021) strains. Strains were grown in liquid MMG at 37°C for 14 h (designated as time point “0”) and transferred into liquid MMG (G; non-inducing) or liquid MMT (T; inducing). Samples were collected at designated time points after the transfer. (C) Positions of putative AREs (5′-CATTCY-3′, indicated by the black oval-shape) in the promoter regions of *velB* and *vosA*. Black lines show the probes that cover ARE. (D) EMSA using the GST-AbaA fusion protein and the radiolabeled probes. Non-radioactive WT or mutant probes were used as competitors and added in 5 to 25 fold molar excess. f.p. = free probe.

### Eletrophoretic Mobility Shift Assay (EMSA)

The cDNA fragment of *abaA* encoding amino acids 1–588 with the ATTA domain was cloned into pGEX-5X-1 (GE Healthcare). The resulting plasmid (pHSE3) was introduced into *E. coli* BL21 (DE3) (Stratagene). The GST fusion protein expression and purification was carried out following the manufacturer’s instruction. For concentration and buffer exchange, Amicon Ultra Centrifilter Unit (Milllipore) was used. BCA Protein Assay Kit (Pierce) was used to estimate protein concentration. EMSA was carried out as described [27]–[29]. Probes for EMSA were generated by annealing two single-stranded reverse-complementary oligonucleotides. The ds oligonucleotides were labeled with γ-^32^P-ATP (PerkinElmer) using T4 polynucleotide kinase (Promega). After labeling, the probes were purified by Sephadex G-25 spin columns. Binding reactions were performed in a 24 µl reaction volume containing 6% glycerol, 12 mM HEPES-KOH [pH 7.6], 4 mM Tris-HCl [pH 8.0], 1 mM EDTA [pH 8.0], 0.5 mM DTT, 0∼12 µg poly(dI-dC), ∼10 ng DNA probe and appropriate amounts of the purified GST::AbaA protein. The reactions were done at RT for 20 min. The complexes were resolved on a 6% polyacrylamide gel (29∶1 crosslinking) with 0.5% TBE running buffer at 100 V for 1 h. The gel was dried down under vacuum to three layers Whatman 3 MM filter paper. Autoradiography was performed at −80°C with Kodak XAR film.

### Purification of *in vivo* VelB Interacting Proteins

For sample preparation, conidia and ascospores from two and seven day grown colonies, respectively, of WT and THS 20.1 (Δ*velB*; *velB*::3xFLAG) strains were collected and suspended in cold PBS with 0.02% Triton X-100. The spores were sonicated for 300 sec (50% pulse) on ice to remove rodlets, and centrifuged for 10 min at 3,000 rpm and the supernatants were removed. For preparation of the protein samples from the mycelium, conidia (5×10^5^ conidia/ml) of WT and THS 20.1 strains were inoculated in 500 ml of liquid MM and incubated for 18 h at 37°C. Mycelial samples were collected, squeeze dried and stored at −80°C. Prepared spores or mycelial samples were broken by a mini-bead beater for 2 cycles (1 min homogenization with 10 min sitting on ice) and centrifuged in a microcentrifuge for 15 min at 15,000 rpm at 4°C. The supernatant was incubated with anti-FLAG M2 affinity gel (A2220, Sigma). The agarose beads were collected by centrifugation and washed three times. The sample was boiled for 5 min and separated by SDS–PAGE and visualized by Coomassie Blue. Each stained band was cut out and subjected to mass spectrometry.

### Nano-LC-ESI-MS/MS and Protein Identifications

All MS/MS experiments for peptide identification were performed using a nano-LC-MS system consisting of an Ultimate HPLC system (Dionex) and Q-TOF mass spectrometer (Micromass, Manchester, UK) equipped with a nano-ESI source, as described previously [30]. Briefly, for each sample, 10 µl was loaded by the autosampler onto a C18 trap column (id 300 mm, length 5 mm, particle size 5 µm; LC Packings) for desalting and concentrating at a flow rate of 20 µl/min. Then the trapped peptides were separated on a 100-mm homemade microcapillary column composed of C18 (Aqua; particle size, 5 µm) packed into 75-µm silica tubing. The mobile phases, A and B, were composed of 0 and 80% acetonitrile, respectively, each containing 0.1% formic acid. The gradient began with 5% B for 15 min; ramped to 20% B over 3 min, to 60% over 45 min, and to 95% over 2 min; and remained at 95% B for another 7 min. The column was equilibrated with 5% B for 10 min before the next run. The voltage applied to produce an electrospray was 2.5 kV. Argon was introduced as a collision gas at a pressure of 10 psi. The three most abundant MS ions were selected by data-dependent peak selection. The previously fragmented ions were excluded for 60 s. The proteins were identified by searching fungi subset (219981 entries) of the National Center for Biotechnology Information (NCBI) protein databases (20100312) using the MASCOT 2.0 search algorithm (Matrix Science). The general parameters for a search were considered to allow a maximum of one missed cleavage, the modifications of N-terminal Gln to pyroGlu, oxidation of methionine, acetylation of protein N terminus, carbamidomethylation of cysteine, and acrylamide modified cysteine. A peptide charge state of +2 or 3, and peptide/fragment mass tolerance of ±0.5 Da were used for the MS/MS ion search. Probability based MASCOT scores were estimated by comparison of search results against estimated random match population and reported as −10*Log(P) where P is the absolute probability. The significance threshold was set at p<0.05.

**Figure 7:**
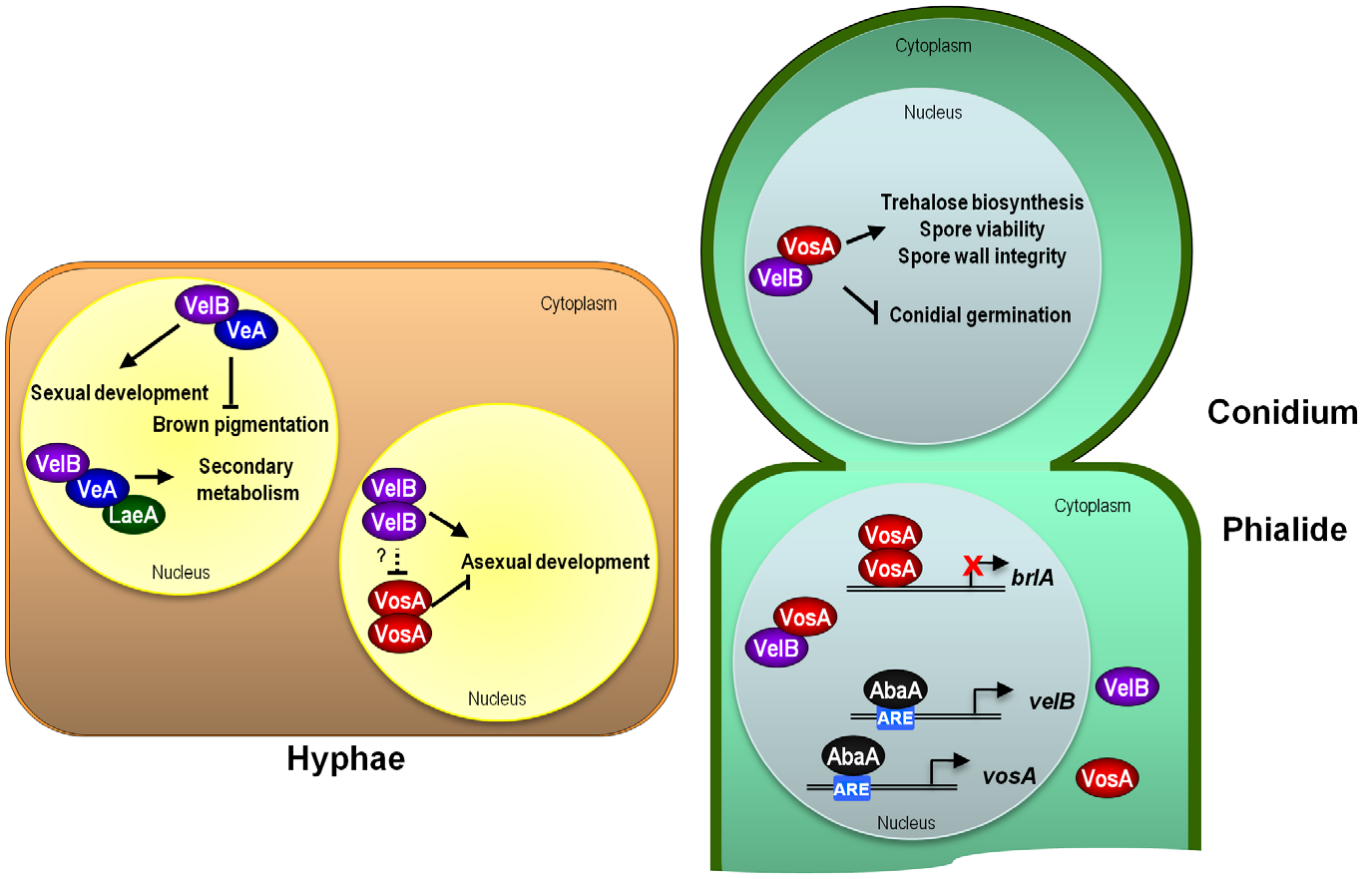
Proposed model for VelB. The cell-type specific roles of the *velvet* complexes in *A. nidulans* are indicated. In vegetative cells (hyphae), VelB functions as a positive regulator of asexual development, whereas VosA represses conidiation. The VelB-VeA heterodimer controls sexual development and forms the VelB-VeA-LaeA trimeric complex that affects secondary metabolism. During conidiogenesis (in phialide) AbaA directly binds to AREs present in the *velB*/*vosA* promoters and regulates *velB*/*vosA* mRNA expression. VosA may bind to the promoter region of *brlA* and negatively controls of *brlA* expression. In conidium, the VelB-VosA complex localizes in the nucleus and plays an essential role in the completion of conidiogenesis, and controlling spore maturation, dormancy and germination.

### Microscopy

The colony photographs were taken by using a Sony digital camera (DSC-F828). Photomicrographs were taken using a Zeiss M^2^ BIO microscope equipped with AxioCam and AxioVision digital imaging software (Zeiss).

### Statistical Analysis

Statistical differences between WT and mutant strains were evaluated with student’s unpaired *t*-test (2-tailed). Mean ± SD are shown. P values <0.05 were considered significant.

## Results

### Developmental Defects Caused by the Lack of VelB

To further investigate the roles of *velB*, we generated *velB* deletion (Δ*velB*) and complemented strains and compared their phenotypes. As shown in Fig. 1A, Δ*velB* strains produced highly elevated levels of brown pigment(s). In the submerged culture, dark brown pigment(s) accumulated in the Δ*velB* hyphae and was detected in liquid medium (data not shown). The *velB* deletion mutant also exhibited reduced and delayed conidiation when point inoculated on solid medium (Fig. 1A). When 10^5^ conidia were inoculated onto solid medium and incubated for 2 days, the Δ*velB* mutant produced a reduced number of conidia (Fig. 1C); about a tenfold decrease in the yield of conidia compared to WT (Fig. 1B, 1D). Essentially, the same results were observed in the *velB* deletion strains with the *veA1* allele (data not shown).

To correlate phenotypic changes caused by deletion of *velB* with the molecular events, we examined the mRNA levels of *brlA*, *abaA* and *vosA* in WT and Δ*velB* strains grown under conditions that induce asexual development (Fig. 1E). In WT, accumulation of *brlA* mRNA was detectable at 6 h post developmental induction and decreased after 24 h. In the Δ*velB* mutant, however, *brlA* mRNA started to accumulate at 12 h and stayed at high levels even after 24 h. Levels of *abaA* mRNA decreased and delayed in the Δ*velB* mutant compared to WT, too. While the short transcript of *vosA* started to accumulate at 24 h and the long transcript of *vosA* accumulated at 48 h in WT, neither long or short transcripts accumulated at 24 h and 48 h after induction of development in the Δ*velB* mutant (Fig. 1F). These results indicate that VelB is necessary for the proper progression of conidiation.

Previous study reported that the Δ*velB* mutant failed to form sexual fruiting bodies in the light and dark conditions [31]. We examined whether the deletion of *velB* affected the expression patterns of key sexual developmental genes including *mutA*, *nosA*, *rosA* and *nsdD* in WT and Δ*velB* strains grown under the conditions that preferentially induce sexual development (Fig. 1G, 1H) [32]–[35]. As shown in Fig. 1H, the deletion of *velB* caused about 24 h delayed (and reduced) accumulation of *mutA* (α-1,3-glucanase) that is associated with Hülle cells [32]. For *rosA* (repressor of sexual development; [33]) transcript, WT showed low level accumulation of its mRNA at 0∼12 h, which then decreased after 24 h post developmental induction. However, the Δ*velB* mutant continued to exhibit (high level) accumulation of *rosA* mRNA even at 24 h post developmental induction, i.e., ∼12 h delay in turning off *rosA* expression compared to WT. During early sexual developmental induction (0∼12 h), levels of *nosA* (number of sexual spores; [34]) mRNA slightly decreased in the Δ*velB* mutant compared to WT. Levels of *nsdD* (never in sexual development; [35]) mRNA were not distinctly different between WT and Δ*velB* strains. These results indicate that VelB is required for proper and timely expression of *mutA*/*rosA*/*nosA* in *A. nidulans*, which is critical for the development of Hülle cells and cleistothecia.

### VelB is Conditionally Sufficient to Enhance Conidiation

We then generated the *velB* overexpression mutant by fusing the *velB* ORF (open reading frame) with the inducible *alcA* promoter [36]. Under non-inducing conditions, there were no differences between WT and overexpression of *velB*. When point inoculated under inducing conditions, overexpression of *velB* with the *veA^+^* or *veA1* allele induced a two-fold increase of asexual spore production compared to WT (Fig. 2A, 2B). We then tested whether VelB has a direct activating potential on *brlA* by examining mRNA levels of *brlA* in OE*velB* strains grown in liquid submerged culture, where neither WT nor OE*velB* strains developed conidiophores (not shown). As shown in Fig. 2C, OE*velB* was not sufficient to induce the activation of *brlA*, indicating that VelB is necessary, and conditionally sufficient for activating conidiation in *A. nidulans*.

### VelB Negatively Controls Germination of Conidia

To test the role of *velB* in spore germination, we examined germination rates of conidia of WT, Δ*velB* and complemented strains in the presence of 1% glucose. At 4 h of incubation, germination rates of WT, Δ*velB* and complemented strains were about 0%, 60% and 0%, respectively. All the Δ*velB* conidia formed germ tubes by 7 h, whereas all conidia of WT and complemented strains germinated by 8 h (Fig. 3A). In further tests, conidia of WT, Δ*velB* and complemented strains were inoculated onto solid media without an external carbon source. At 5 h after inoculation, some of the Δ*velB* mutant conidia began to form germ tubes but these were not detectable in WT and complemented strains (Fig. 3B). These results indicate that VelB is negatively associated with conidial germination.

Previous studies suggested that GanB(α)-mediated signal transduction plays a crucial role in controlling germination and pigmentation via cAMP/PKA signaling pathway, and is attenuated by the regulator of G-protein signaling (RGS) protein RgsA [24], [37]–[39]. Similar to Δ*rgsA*, a dominant activating GanB mutant allele caused enhanced accumulation of brown pigment(s) and increased conidial germination rates in the absence of a carbon source [24], [37]. To study a potential relationship between VelB and the GanB signaling pathway, we generated the Δ*velB* Δ*ganB* double mutant and compared its phenotypes including brown pigment production and conidial germination with the Δ*velB* mutant. The Δ*velB* Δ*ganB* double mutant showed reduced accumulation of brown pigment compared to the Δ*velB* single mutant. Similar to Δ*velB*, however, the Δ*velB* Δ*ganB* double mutant exhibited reduced radial growth. As shown in Fig. 3C, although no WT and Δ*ganB* conidia germinated, about 60% of the Δ*velB* Δ*ganB* double mutant conidia could germinate at 4 h of incubation. Taken together, these results suggest that, VelB may play an independent role to GanB in brown-pigment(s) production, but function downstream of GanB-mediated signaling in regulating conidial germination (Fig. 3D).

### Inter-dependence of VelB and VosA

Previous studies showed that both VelB and VosA are needed for sporogenesis and trehalose biogenesis in *A. nidulans* [16]. To examine the potential inter-dependent relationship between VelB and VosA, we generated the *vosA* and *velB* double deletion mutant and checked the spore viability, trehalose amount and mRNA levels of *tpsA* in WT and mutants. When point inoculated on solid medium (Fig. 4A), the Δ*velB* Δ*vosA* double mutant showed reduced conidiation compared to the WT and Δ*vosA* single mutant, but increased the number of conidia compared to the Δ*velB* single mutant. These results suggest that VelB (activator) and VosA (repressor) provide independent inputs to the final levels of asexual development and the double deletion results in an inter-mediate phenotype (Fig. 4B). The conidia of both single and double mutants displayed severe viability defects (Fig. 4C). We measured trehalose amounts in 2 day old fresh conidia and found that trehalose was undetectable in the double deletion mutant (Fig. 4D). Northern blot of *tpsA*, which is associated with the synthesis of trehalose, revealed that both single and double deletion mutants exhibited reduced *tpsA* transcript levels in conidia (Fig. 4E). We also examined the tolerance of the spores to oxidative and UV stresses and found that the Δ*velB* Δ*vosA* mutant conidia, similar to the Δ*velB* or Δ*vosA* single mutant conidia, were more sensitive to oxidative and UV stresses compared to WT conidia (not shown). Moreover, we found that the absence of *vosA*, similar to Δ*velB*, caused increased conidial germination rates in the presence or absence of carbon source (not shown). We also observed that germination rates of the Δ*velB* Δ*vosA* mutant conidia were similar to the Δ*velB* or Δ*vosA* single mutant conidia regardless of the presence or absence of an external carbon source. Taken together, these results suggest that VelB and VosA play an inter-dependent (not additive) role in sporogenesis, trehalose biogenesis and controlling conidial germination.

### VelB Primarily Interacts with VosA in Asexual and Sexual Spores

VelB has been shown to form not only the VelB-VeA-LaeA heterotrimeric complex, which coordinates secondary metabolism and development, but also the VelB-VosA heterodimer, which may be the functional unit of trehalose biosynthesis and spore maturation [15], [16]. The C-terminal region of VelB interacts with VeA or VosA [15]. Genetic data for the inter-dependent role of VelB and VosA in spores, and high accumulation of both *vosA* and *velB* transcripts in asexual and sexual spores [15], led us to hypothesize that VelB mainly interacts with VosA and forms the functional hetero-dimeric complex in the spores. To test this, we first generated the strains expressing the VelB protein fused with a 3xFLAG C-terminal tag in the Δ*velB* background in *A. nidulans* and examined the levels of the VelB protein throughout the life cycle. Through Western blot with FLAG antibody, we found that the VelB::3XFLAG protein is clearly detectable during vegetative growth and early asexual development, and accumulates at high levels in conidia (Fig. S1). We confirmed that the VosA protein highly accumulates in conidia, too (Fig. S1). We then collected conidia and ascospores from 2 and 7 day, respectively, grown colonies of the strain expressing a functional VelB::3xFLAG fusion driven by the *velB* native promoter and identified the VelB interacting protein(s). We found that the VelB::3XFLAG immuno-pull-down predominantly isolated VosA and VelB, but not VeA or LaeA, in both asexual and sexual spores (Fig. 5 A&B). We further examined the VelB interacting protein(s) in hyphae and found that VelB::3XFLAG isolates VeA, but not VosA (Fig. 5C). These results suggest that VelB interacts with VeA (hyphae) or VosA (spores), and plays a differential role in controlling various biological processes.

### AbaA Regulates *velB* and *vosA* Expression

During asexual development, mRNA levels of *velB* and *vosA* increase during the formation and maturation of conidia [13], [15]. Through sequence analysis, we found that three and one AbaA response elements (AREs, 5′-CATTCY-3′) are located at the promoter and at N-terminal ORF regions, respectively, of *velB*, and hypothesized that AbaA directly binds and regulates *velB* (and *vosA*) expression in the late asexual development phases. To test this hypothesis, levels of *velB* mRNA were examined in various mutants carrying the *brlA42*, *abaA14*, *wetA6*. In the *brlA42* and *abaA14* mutants, *velB* mRNA accumulation decreased compared to WT, but in the *wetA6* mutant, levels of *velB* mRNA were comparable to WT, suggesting that *brlA* and/or *abaA* are required for *velB* mRNA accumulation (Fig. 6A). To further test a potential direct activating role of BrlA and/or AbaA in *velB* expression, we examined levels of *velB* mRNA in the *alcA*(p)::*brlA*, *alcA*(p)::*brlA abaA14* and *alcA*(p)::*abaA* mutants. As shown in Fig. 6B, *velB* mRNA highly accumulated at 6 h upon *abaA* overexpression. Levels of *velB* mRNA slightly increased at 24 h after *brlA* overexpression, which was dependent on the WT abaA allele, as *velB* mRNA was not induced in the *alcA*(p)::*brlA abaA14* mutant. These results indicate that AbaA, not BrlA, is necessary and sufficient for *velB* mRNA expression.

We then investigated whether AbaA directly binds to the *velB* and *vosA* promoter regions by EMSA. We designed and used two probes covering AREs in the *velB* promoter and one probe covering ARE in the *vosA* promoter (Fig. 6C). As shown in Fig. 6D, the GST-AabA fusion protein binds to all three probes containing ARE whereas the GST protein alone failed to bind to these probes. To further corroborate these AbaA-DNA interactions, we designed another probe with the mutated ARE motif. The GST-AbaA fusion protein could not bind to these mutated probes (data not shown). Moreover, only the unlabeled WT probes, but not mutated probes, competed during the complex formation with the labeled WT ARE probes (Fig. 6C). These results demonstrate that AbaA directly binds to the promoter regions of *velB/vosA* and regulates *velB/vosA* mRNA expression.

## Discussion

The *velvet* proteins are fungi-specific multifunctional regulators that control development and secondary metabolism in various filamentous and dimorphic fungi [14], [40]. In *A. nidulans*, VelB plays an essential role in sexual development, the mycotoxin sterigmatocystin production and trehalose biosynthesis [15], [16]. VelB homologues have been characterized in many fungi and are reported to regulate: sporulation and secondary metabolite production in *Fusarium fujikuroi* [41]; production of the trichothecene and zearalenone mycotoxins, pathogenicity, and sexual reproduction in *Fusarium graminearum* [42]; cell morphology and spore formation in *Histoplasma capsulatum* [43]; trehalose biosynthesis and conidiation in *Aspergillus fumigatus* (Park and Yu, unpublished); and production of the carcinogenic mycotoxin aflatoxin and formation of sclerotia in *Aspergillus flavus* (Park, Lovendahl and Yu, unpublished).

Our characterization of VelB in asexual development and sporogenesis indicates that VelB acts as a positive regulator of asexual development, and an essential component for trehalose biogenesis in spores. Previous studies suggested that the VosA-VelB heterodimer represses asexual development during vegetative growth in liquid culture and early asexual development in the dark [16]. Unlike Δ*vosA* strains, however, the Δ*velB* mutant (i) cannot produce conidiophores in liquid submerged culture; (ii) produces a lower number of asexual spores than WT; and (iii) exhibits lowered *brlA* mRNA accumulation in early phase of asexual development. In addition, unlike the Δ*vosA* single mutant, the Δ*velB* Δ*vosA* double mutant does not develop conidiophores in liquid submerged culture, i.e., VelB is epistatic to VosA. These results suggest that VelB is required for the hyper-conidiation in the absence of *vosA*, further supporting the proposed positive role of VelB in asexual development. Taken together, we propose that the VelB monomer or homo-dimer functions as a positive regulator of asexual development, whereas the VosA homo-dimer plays a negative regulatory role in conidiation during vegetative growth and the early phase of conidiophore formation (see Fig. 7).

VelB participates in the formation of multiple *velvet* complexes including VelB-VeA and VelB-VeA-LaeA that play differential roles in sexual development and secondary metabolism [14], [15]. Our studies suggest that in hyphae VelB predominantly interacts with VeA and may form a VeA-VelB complex (Fig. 5C). As shown in Figs 1&2, however, VelB’s function in asexual spore formation does not require the physical interaction with VeA, as VeA1 cannot physically interact with VelB (Bayram *et al*., 2008). These data suggest that VelB and VeA may play a redundant role in controlling asexual spore formation, but an inter-dependent role in sexual development by forming a VelB-VeA functional unit [15]. Similar to *veA*, moreover, the deletion of *velB* resulted in reduced colony size and elevated accumulation of brown pigment(s). The phenotypes, accumulation of brown pigment(s) and reduced colony growth, of the Δ*veA* or Δ*velB* single mutant were similar to those of the Δ*veA* Δ*velB* double mutant, suggesting that VeA and VelB also function inter-dependently to regulate brown pigmentation and colony growth. In addition, VeA and VelB control secondary metabolism by forming trimeric complexes with the master regulator of secondary metabolism LaeA [15].

It appears that, in addition to activating conidiation, VelB is required for proper control of conidial germination. Trehalose is not only required for the acquisition of tolerance to various stresses but also utilized in glycolysis, spore maturation and germination [11], [44]. Trehalose is rapidly degraded during conidial germination and the absence of the trehalose-6-phosphate synthase gene caused delayed conidial germination in aspergilli [11], [45]–[47]. However, exogenous trehalose supplemented in the medium had no effects on rescuing the delayed conidial germination caused by the deletion of trehalose-6-phosphate synthase in *A. fumigatus*, suggesting that trehalose-6-phosphate synthase genes, but not trehalose, may play an additional role in conidial germination [47]. In our present study, despite the lack of trehalose in the *velB* deletion mutant conidia, germination rates of Δ*velB* conidia were increased (Fig. 3). These suggest the possibility of VelB playing a more complex role in governing spore germination, which may be associated the GanB(α)-mediated signal transduction. We propose that VelB functions as a negative regulator of conidial germination acting downstream of GanB-mediated signaling (Fig. 3D).

Previous studies demonstrated that the absence of *velB* or *vosA* resulted in the lack of trehalose in conidia and shortened viability of spores, and that the VosA-VelB complex is formed in the nucleus. These suggest that the VelB-VosA complex is the functional unit governing conidial maturation [13], [16]. Results of our double mutant analyses are generally in agreement with the proposed inter-dependent role of VelB and VosA in spore viability, trehalose biosynthesis and conidial germination. Our data further demonstrate that VelB predominantly interacts with VosA in the asexual and sexual spores (Fig. 5). Taken together, we confirmed that the VelB-VosA complex is a major unit playing a vital role in controlling spore maturation (trehalose biogenesis and cell wall completion), long-term viability, dormancy and germination.

BrlA is mainly localized in vesicles metulae, and phialides, and young spores and regulates conidiogenesis during the early phase of conidiation [48], [49]. However, *brlA* mRNA has not been detected in mature spores [50]. To complete sporogenesis turning off the expression of *brlA* is crucial for fungal spores. Uncontrolled production of *brlA* may inhibit growth of *A. nidulans*, likely causing a generalized metabolic shutdown, leading to an inability of cells to acquire nutrients from the growth medium. [51]. Previous study demonstrated that VosA exerts negative feedback regulation of conidiation, i.e., turns off *brlA* expression after spore formation, and the deletion of *vosA* resulted in high level accumulation of *brlA* mRNA in conidia [13]. Our preliminary genome wide analyses of *in vivo* VosA-DNA interactions indicate that the promoter regions of *brlA* are specifically enriched, suggesting that VosA may directly bind to the promoter of *brlA* and repress *brlA* expression in phialides and/or conidia.

Based on these data, we propose a genetic model depicting the roles and regulation of VelB and VosA during conidiogenesis. VelB is a multifunctional developmental regulator playing a pivotal role throughout the life cycle of *A. nidulans*. The interaction between VelB and its partner proteins (VelB, VeA or VosA) is time and/or cell-type specific and the multiple VelB-related complexes play differential roles in controlling fungal development and sporogenesis (see model in Fig. 7). During conidiogenessis, both *velB* and *vosA* are activated by AbaA in the vesicle and phialide [13]. High level production of VelB and VosA in developing cells would induce the formation of the VosA-VosA and VelB-VosA complexes. Genetic data in conjunction with VosA’s localization in metulae, phialides and conidia suggest that the VosA monomer or homo-dimer turns off *brlA* expression in conidia [13]. The VelB-VosA hetero-dimer activates focal trehalose biogenesis in spores, controls spore wall integrity, and negatively regulates precocious conidial germination (Fig. 7). Further in-depth analyses of the roles of the individual *velvet* proteins and complexes, and the mechanisms of *velvet*-mediated regulation are in progress and will provide new insights into fungal development and secondary metabolism.

## Supporting Information

Figure S1 **Levels of the VelB and VosA proteins throughout the lifecycle of** ***A. nidulans*** **.** Western blot for the VelB::3xFLAG and VosA::3xFLAG fusion proteins in *velB*(p)::VelB::3xFLAG (THS20.1) and *vosA*(p)::VosA::3xFLAG (THS28.1) strains, respectively. These fusion proteins were detected by anti-FLAG antibody. Protein crude extracts (10 µg) were loaded in each lane. Conidia (asexual spores) were indicated as C. The numbers indicate the time (hours) after incubation in liquid MMG (Vegetative) and solid MMG inducing asexual development (Asexual).
(TIF)

## References

[1] Latge JP (1999) *Aspergillus fumigatus* and aspergillosis. Clin Microbiol Rev 12: 310–350. 10194462 10.1128/cmr.12.2.310 PMC88920

[2] Yu J-H, Keller N (2005) Regulation of secondary metabolism in filamentous fungi. Annu Rev Phytopathol 43: 437–458. 16078891 10.1146/annurev.phyto.43.040204.140214

[3] Calvo AM, Wilson RA, Bok JW, Keller NP (2002) Relationship between secondary metabolism and fungal development. Microbiol Mol Biol Rev 66: 447–459, table of contents. 10.1128/MMBR.66.3.447-459.2002 PMC120793 12208999

[4] Adams TH, Wieser JK, Yu J-H (1998) Asexual sporulation in *Aspergillus nidulans* . Microbiol Mol Biol Rev 62: 35–54. 9529886 10.1128/mmbr.62.1.35-54.1998 PMC98905

[5] Casselton L, Zolan M (2002) The art and design of genetic screens: filamentous fungi. Nat Rev Genet 3: 683–697. 12209143 10.1038/nrg889

[6] Yu J-H (2010) Regulation of development in *Aspergillus nidulans* and *Aspergillus fumigatus* . Mycobiology 38: 229–237. 23956662 10.4489/MYCO.2010.38.4.229 PMC3741515

[7] Ni M, Gao N, Kwon N-J, Shin K-S, Yu J-H (2010) Regulation of *Aspergillus* Conidiation. Cellular and Molecular Biology of Filamentous Fungi: 559–576.

[8] Sewall TC, Mims CW, Timberlake WE (1990) *abaA* controls phialide differentiation in *Aspergillus nidulans* . Plant Cell 2: 731–739. 2152124 10.1105/tpc.2.8.731 PMC159926

[9] Adams TH, Timberlake WE (1990) Upstream elements repress premature expression of an *Aspergillus* developmental regulatory gene. Mol Cell Biol 10: 4912–4919. 2117702 10.1128/mcb.10.9.4912-4919.1990 PMC361108

[10] Andrianopoulos A, Timberlake WE (1994) The *Aspergillus nidulans abaA* gene encodes a transcriptional activator that acts as a genetic switch to control development. Mol Cell Biol 14: 2503–2515. 8139553 10.1128/mcb.14.4.2503-2515.1994 PMC358618

[11] Fillinger S, Chaveroche MK, van Dijck P, de Vries R, Ruijter G, et al (2001) Trehalose is required for the acquisition of tolerance to a variety of stresses in the filamentous fungus *Aspergillus nidulans* . Microbiology 147: 1851–1862. 11429462 10.1099/00221287-147-7-1851

[12] Paul MJ, Primavesi LF, Jhurreea D, Zhang Y (2008) Trehalose metabolism and signaling. Annu Rev Plant Biol 59: 417–441. 18257709 10.1146/annurev.arplant.59.032607.092945

[13] Ni M, Yu J-H (2007) A novel regulator couples sporogenesis and trehalose biogenesis in *Aspergillus nidulans* . PLoS One 2: e970. 17912349 10.1371/journal.pone.0000970 PMC1978537

[14] Bayram O, Braus GH (2012) Coordination of secondary metabolism and development in fungi: the *velvet* family of regulatory proteins. FEMS Microbiol Rev 36: 1–24. 21658084 10.1111/j.1574-6976.2011.00285.x

[15] Bayram O, Krappmann S, Ni M, Bok JW, Helmstaedt K, et al (2008) VelB/VeA/LaeA complex coordinates light signal with fungal development and secondary metabolism. Science 320: 1504–1506. 18556559 10.1126/science.1155888

[16] Sarikaya Bayram O, Bayram O, Valerius O, Park HS, Irniger S, et al (2010) LaeA control of *velvet* family regulatory proteins for light-dependent development and fungal cell-type specificity. PLoS Genet 6: e1001226. 21152013 10.1371/journal.pgen.1001226 PMC2996326

[17] Kafer E (1977) Meiotic and mitotic recombination in *Aspergillus* and its chromosomal aberrations. Adv Genet 19: 33–131. 327767 10.1016/s0065-2660(08)60245-x

[18] Seo JA, Guan Y, Yu JH (2003) Suppressor mutations bypass the requirement of *fluG* for asexual sporulation and sterigmatocystin production in *Aspergillus nidulans* . Genetics 165: 1083–1093. 14668366 10.1093/genetics/165.3.1083 PMC1462808

[19] Seo J-A, Han K-H, Yu J-H (2004) The *gprA* and *gprB* genes encode putative G protein-coupled receptors required for self-fertilization in *Aspergillus nidulans* . Mol Microbiol 53: 1611–1623. 15341643 10.1111/j.1365-2958.2004.04232.x

[20] Yu JH, Hamari Z, Han KH, Seo JA, Reyes-Dominguez Y, et al (2004) Double-joint PCR: a PCR-based molecular tool for gene manipulations in filamentous fungi. Fungal Genet Biol 41: 973–981. 15465386 10.1016/j.fgb.2004.08.001

[21] Szewczyk E, Nayak T, Oakley CE, Edgerton H, Xiong Y, et al (2006) Fusion PCR and gene targeting in *Aspergillus nidulans* . Nat Protoc 1: 3111–3120. 17406574 10.1038/nprot.2006.405

[22] Osmani AH, May GS, Osmani SA (1999) The extremely conserved *pyroA* gene of *Aspergillus nidulans* is required for pyridoxine synthesis and is required indirectly for resistance to photosensitizers. Journal of Biological Chemistry 274: 23565–23569. 10438537 10.1074/jbc.274.33.23565

[23] Yelton MM, Hamer JE, Timberlake WE (1984) Transformation of *Aspergillus nidulans* by using a *trpC* plasmid. Proc Natl Acad Sci U S A 81: 1470–1474. 6324193 10.1073/pnas.81.5.1470 PMC344858

[24] Han K-H, Seo J-A, Yu J-H (2004) Regulators of G-protein signalling in *Aspergillus nidulans*: RgsA downregulates stress response and stimulates asexual sporulation through attenuation of GanB (Galpha) signalling. Mol Microbiol 53: 529–540. 15228532 10.1111/j.1365-2958.2004.04163.x

[25] d’Enfert C, Fontaine T (1997) Molecular characterization of the *Aspergillus nidulans* treA gene encoding an acid trehalase required for growth on trehalose. Mol Microbiol 24: 203–216. 9140977 10.1046/j.1365-2958.1997.3131693.x

[26] Ni M, Rierson S, Seo JA, Yu JH (2005) The pkaB gene encoding the secondary protein kinase A catalytic subunit has a synthetic lethal interaction with pkaA and plays overlapping and opposite roles in Aspergillus nidulans. Eukaryot Cell 4: 1465–1476. 16087751 10.1128/EC.4.8.1465-1476.2005 PMC1214532

[27] Dutton JR, Johns S, Miller BL (1997) StuAp is a sequence-specific transcription factor that regulates developmental complexity in *Aspergillus nidulans* . EMBO J 16: 5710–5721. 9312029 10.1093/emboj/16.18.5710 PMC1170202

[28] Hellman LM, Fried MG (2007) Electrophoretic mobility shift assay (EMSA) for detecting protein-nucleic acid interactions. Nat Protoc 2: 1849–1861. 17703195 10.1038/nprot.2007.249 PMC2757439

[29] Park BC, Park YH, Park HM (2003) Activation of *chsC* transcription by AbaA during asexual development of *Aspergillus nidulans* . FEMS Microbiol Lett 220: 241–246. 12670687 10.1016/S0378-1097(03)00120-4

[30] Kim JY, Song HJ, Lim HJ, Shin MG, Kim JS, et al (2008) Platelet factor-4 is an indicator of blood count recovery in acute myeloid leukemia patients in complete remission. Mol Cell Proteomics 7: 431–441. 17998245 10.1074/mcp.M700194-MCP200

[31] Bayram O, Krappmann S, Ni M, Bok JW, Helmstaedt K, et al (2008) VelB/VeA/LaeA complex coordinates light signal with fungal development and secondary metabolism. Science 320: 1504–1506. 18556559 10.1126/science.1155888

[32] Wei H, Scherer M, Singh A, Liese R, Fischer R (2001) *Aspergillus nidulans* alpha-1,3 glucanase (mutanase), *mutA*, is expressed during sexual development and mobilizes mutan. Fungal Genet Biol 34: 217–227. 11728159 10.1006/fgbi.2001.1303

[33] Vienken K, Scherer M, Fischer R (2005) The Zn(II)2Cys6 putative *Aspergillus nidulans* transcription factor repressor of sexual development inhibits sexual development under low-carbon conditions and in submersed culture. Genetics 169: 619–630. 15520269 10.1534/genetics.104.030767 PMC1449130

[34] Vienken K, Fischer R (2006) The Zn(II)2Cys6 putative transcription factor NosA controls fruiting body formation in *Aspergillus nidulans* . Mol Microbiol 61: 544–554. 16780567 10.1111/j.1365-2958.2006.05257.x

[35] Han K-H, Han K-Y, Yu J-H, Chae K-S, Jahng K-Y, et al (2001) The *nsdD* gene encodes a putative GATA-type transcription factor necessary for sexual development of *Aspergillus nidulans* . Mol Microbiol 41: 299–309. 11489119 10.1046/j.1365-2958.2001.02472.x

[36] Gwynne DI, Buxton FP, Sibley S, Davies RW, Lockington RA, et al (1987) Comparison of the cis-acting control regions of two coordinately controlled genes involved in ethanol utilization in *Aspergillus nidulans* . Gene 51: 205–216. 3297923 10.1016/0378-1119(87)90309-x

[37] Chang MH, Chae KS, Han DM, Jahng KY (2004) The GanB Galpha-protein negatively regulates asexual sporulation and plays a positive role in conidial germination in *Aspergillus nidulans* . Genetics 167: 1305–1315. 15280244 10.1534/genetics.103.025379 PMC1470946

[38] Lafon A, Seo J-A, Han K-H, Yu J-H, d’Enfert C (2005) The heterotrimeric G-protein GanB(alpha)-SfaD(beta)-GpgA(gamma) is a carbon source sensor involved in early cAMP-dependent germination in *Aspergillus nidulans* . Genetics 171: 71–80. 15944355 10.1534/genetics.105.040584 PMC1456537

[39] Yu J-H (2006) Heterotrimeric G protein signaling and RGSs in *Aspergillus nidulans* . J Microbiol 44: 145–154. 16728950

[40] Calvo AM (2008) The VeA regulatory system and its role in morphological and chemical development in fungi. Fungal Genet Biol 45: 1053–1061. 18457967 10.1016/j.fgb.2008.03.014

[41] Wiemann P, Brown DW, Kleigrewe K, Bok JW, Keller NP, et al (2010) FfVel1 and FfLae1, components of a *velvet*-like complex in *Fusarium fujikuroi*, affect differentiation, secondary metabolism and virulence. Mol Microbiol 77: 972–994. 20572938 10.1111/j.1365-2958.2010.07263.x PMC2989987

[42] Lee J, Myong K, Kim JE, Kim HK, Yun SH, et al.. (2012) FgVelB globally regulates sexual reproduction, mycotoxin production, and pathogenicity in the cereal pathogen Fusarium graminearum. Microbiology. 10.1099/mic.0.059188-0 22516221

[43] Webster RH, Sil A (2008) Conserved factors Ryp2 and Ryp3 control cell morphology and infectious spore formation in the fungal pathogen *Histoplasma capsulatum* . Proc Natl Acad Sci U S A 105: 14573–14578. 18791067 10.1073/pnas.0806221105 PMC2567189

[44] Elbein AD, Pan YT, Pastuszak I, Carroll D (2003) New insights on trehalose: a multifunctional molecule. Glycobiology 13: 17R–27R. 10.1093/glycob/cwg047 12626396

[45] Kane SM, Roth R (1974) Carbohydrate metabolism during ascospore development in yeast. J Bacteriol 118: 8–14. 4595206 10.1128/jb.118.1.8-14.1974 PMC246633

[46] Shin K-S, Kwon N-J, Yu J-H (2009) Gbetagamma-mediated growth and developmental control in *Aspergillus fumigatus* . Curr Genet 55: 631–641. 19915845 10.1007/s00294-009-0276-4

[47] Al-Bader N, Vanier G, Liu H, Gravelat FN, Urb M, et al (2010) Role of trehalose biosynthesis in *Aspergillus fumigatus* development, stress response, and virulence. Infect Immun 78: 3007–3018. 20439478 10.1128/IAI.00813-09 PMC2897364

[48] Adams TH, Boylan MT, Timberlake WE (1988) *brlA* is necessary and sufficient to direct conidiophore development in *Aspergillus nidulans* . Cell 54: 353–362. 3293800 10.1016/0092-8674(88)90198-5

[49] Aguirre J (1993) Spatial and temporal controls of the *Aspergillus brlA* developmental regulatory gene. Mol Microbiol 8: 211–218. 8316075 10.1111/j.1365-2958.1993.tb01565.x

[50] Boylan MT, Mirabito PM, Willett CE, Zimmerman CR, Timberlake WE (1987) Isolation and physical characterization of three essential conidiation genes from *Aspergillus nidulans* . Mol Cell Biol 7: 3113–3118. 2823119 10.1128/mcb.7.9.3113-3118.1987 PMC367944

[51] Adams TH, Timberlake WE (1990) Developmental repression of growth and gene expression in *Aspergillus* . Proc Natl Acad Sci U S A 87: 5405–5409. 2196567 10.1073/pnas.87.14.5405 PMC54333

[52] Clutterbuck AJ (1969) A mutational analysis of conidial development in *Aspergillus nidulans* . Genetics 63: 317–327. 5366214 10.1093/genetics/63.2.317 PMC1212347

