# Supplementary figures and images for: The Role, Interaction and Regulation of the Velvet Regulator VelB in Aspergillus nidulans
Source: PLoS One. 2012 Sep 25;7(9):e45935. doi: 10.1371/journal.pone.0045935 (PMC3457981; doi:10.1371/journal.pone.0045935)

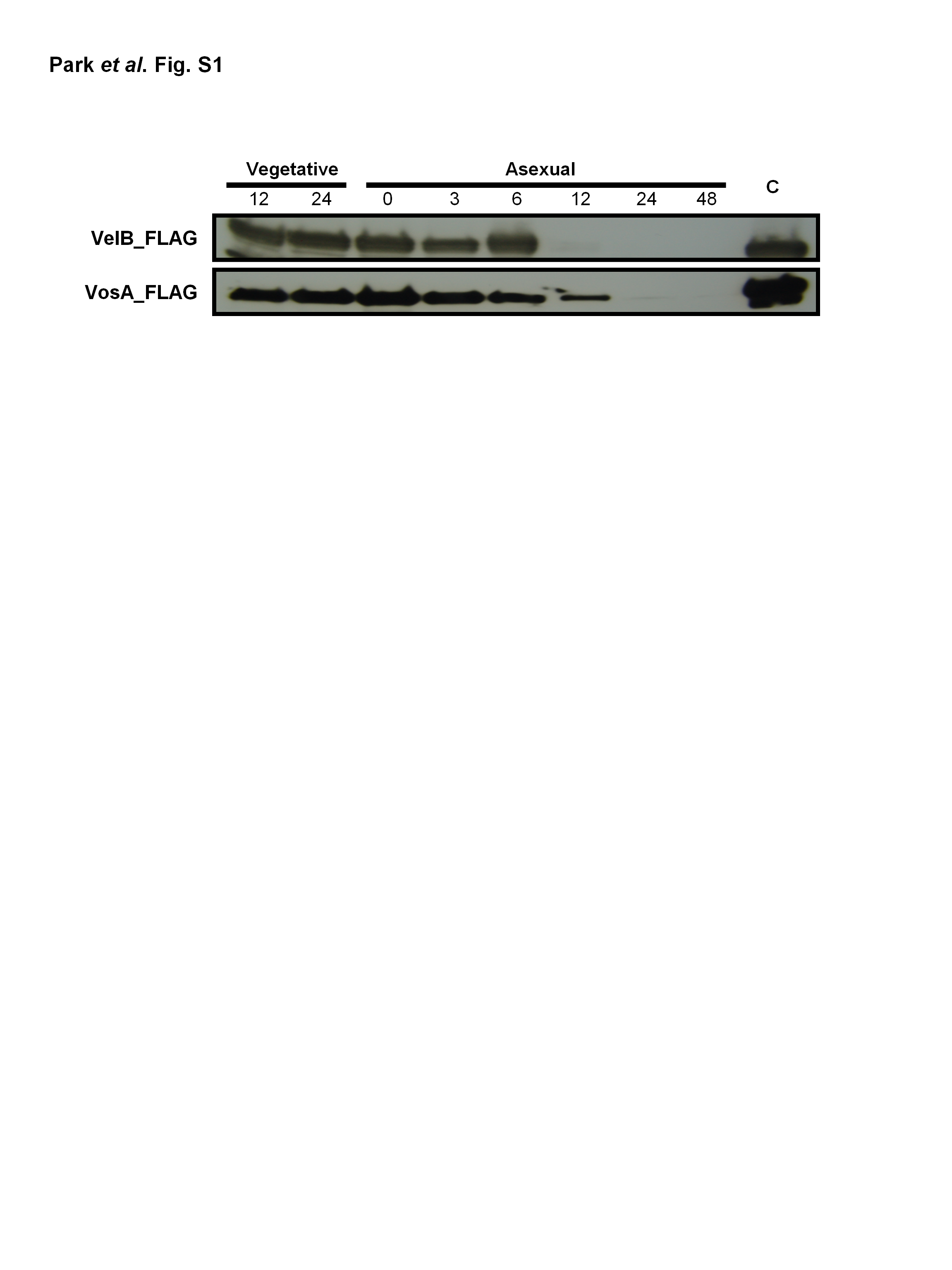

Supplement: Figure S1 — Levels of the VelB and VosA proteins throughout the lifecycle of A. nidulans . Western blot for the VelB::3xFLAG and VosA::3xFLAG fusion proteins in velB(p)::VelB::3xFLAG (THS20.1) and vosA(p)::VosA::3xFLAG (THS28.1) strains, respectively. These fusion proteins were detected by anti-FLAG antibody. Protein crude extracts (10 µg) were loaded in each lane. Conidia (asexual spores) were indicated as C. The numbers indicate the time (hours) after incubation in liquid MMG (Vegetative) and solid MMG inducing asexual development (Asexual). (TIF) [file pone.0045935.s001.tif]
